# Analyses of associations between three positionally cloned asthma candidate genes and asthma or asthma-related phenotypes in a Chinese population

**DOI:** 10.1186/1471-2350-10-123

**Published:** 2009-12-01

**Authors:** Huanyu Zhou, Xiumei Hong, Shanqun Jiang, Hongxing Dong, Xiping Xu, Xin Xu

**Affiliations:** 1 Program for Population Genetics, Harvard School of Public Health, Boston, Massachusetts, USA; 2 Division of Epidemiology and Biostatistics, School of Public Health, University of Illinois at Chicago, Chicago, Illinois, USA; 3 Institute of Biomedicine, Anhui Medical University, Hefei, Anhui, PR China

## Abstract

**Background:**

Six asthma candidate genes, ADAM33, NPSR1, PHF11, DPP10, HLA-G, and CYFIP2, located at different chromosome regions have been positionally cloned following the reported linkage studies. For ADAM33, NPSR1, and CYFIP2, the associations with asthma or asthma-related phenotypes have been studied in East Asian populations such as Chinese and Japanese. However, for PHF11, DPP10, and HLA-G, none of the association studies have been conducted in Asian populations. Therefore, the aim of the present study is to test the associations between these three positionally cloned genes and asthma or asthma-related phenotypes in a Chinese population.

**Methods:**

Two, five, and two single nucleotide polymorphisms (SNPs) in the identified top regions of PHF11, DPP10, and HLA-G, respectively, were genotyped in 1183 independent samples. The study samples were selected based on asthma affectation status and extreme values in at least one of the following three asthma-related phenotypes: total serum immunoglobulin E levels, bronchial responsiveness test, and skin prick test. Both single SNP and haplotype analyses were performed.

**Results:**

We found that DPP10 was significantly associated with bronchial hyperresponsiveness (BHR) and BHR asthma after the adjustment for multiple testing; while the associations of PHF11 with positive skin reactions to antigens and the associations of HLA-G with BHR asthma were only nominally significant.

**Conclusion:**

Our study is the first one to provide additional evidence that supports the roles of DPP10 in influencing asthma or BHR in a Chinese population.

## Background

Six genes, plant homeodomain finger protein 11 (PHF11), dipeptidyl-peptidase 10 (DPP10), histocompatibility antigen, class I, G (HLA-G), ADAM metallopeptidase domain 33 (ADAM33), neuropeptide S receptor 1 (NPSR1, previously GPR154 or GPRA), and cytoplasmic FMR1 interacting protein 2 (CYFIP2), have been identified to be associated with asthma and asthma-related phenotypes following linkage studies [1-6]. These genes span a wide range of functions, which were either unknown or would not have been considered to be implicated in the etiology of asthma before their discovery.

PHF11 was positionally cloned by Zhang *et al*. [1] and has been found to be associated with IgE levels, severe clinical asthma and childhood atopic dermatitis in Caucasian populations [1,7]. A more recent study demonstrated that knockdown of PHF11 expression reduced expression of the T(H)1 - type cytokines IFN-gamma and IL-2 [8]. Allen *et al*. identified DPP10 and showed its associations with asthma in Caucasian populations [2]. Other studies have demonstrated that DPP10 modulated functional properties of Kv4-mediated A-type K+ channels in the central neurons via its intracellular and transmembrane domains [9-11]. Nicolae *et al*. positionally cloned HLA-G and showed that HLA-G was associated with asthma, bronchial hyperresponsiveness (BHR) and atopy in Caucasian populations [3]. HLA-G was expressed in bronchial epithelial cells [3] and may be involved in airway remodeling by inhibiting angiogenesis [12]. ADAM33 was identified by Van Eerdewegh *et al*. [4], and following functional studies have suggested that it might play roles in airway remodeling by promoting angiogenesis [13]. Laitinen *et al*. identified NPSR1 as an asthma candidate gene and reported that levels of the NPSR1-B isoform were increased in airway smooth muscle cells and epithelial cells in asthma patients compared to healthy controls, and NPSR1 was up-regulated in a mouse model of ovalbumin-induced inflammation [5]. CYFIP2 was positionally cloned by Noguchi *et al*. [6] and has been implicated in Rac-1-mediated T cell adhesion [14].

Recently, in studies of childhood asthma, Hersh *et al*. attempted to replicate associations with five positionally cloned asthma susceptibility genes, including ADAM33, DPP10, NPSR1, HLA-G, and PHF11, using both Caucasian and Hispanic families [15]. However, the replication with asthma was only found in NPSR1 and PHF11 in both cohorts, yet for NPSR1, the opposite alleles of SNPs were associated in either cohort, and for PHF11, there was no overlap in the associated SNPs across the two cohorts; no association was found in ADAM33, DPP10 or HLA-G in either cohort. A more recent study was conducted to replicate associations of 93 previously reported asthma candidate genes, including the six positionally cloned genes, with asthma and asthma-related phenotypes using 5,565 individuals (predominantly Caucasian); however, no significant associations were found for these six genes [16]. Actually, the associations of ADAM33, NPSR1, and CYFIP2 with asthma or asthma-related phenotypes have been studied in Chinese or Japanese populations [6,17,18]. However, to our knowledge, there is no further confirmation study conducted for DPP10, HLA-G, and PHF11, and the reported associations in non-Caucasian populations have yet to be examined. In this article, we examined the associations of polymorphisms in the identified top regions of PHF11, DPP10, and HLA-G with asthma and asthma-related phenotypes in a Chinese population, using a population-based study design.

## Methods

### Study Population and Phenotype Definition

In the current study, based on asthma affectation status and extreme values in asthma-related phenotypes, we selected a total of 1183 samples from 3022 families (2752 asthma index families and 270 reference families) that were enrolled from Anqing city in China. A detailed description of study site, subject recruitment, and phenotype measurements has been presented elsewhere previously [19,20]. In brief, 2752 index families, which included both parents and at least 2 offspring who were ≥8 years old and had physician-diagnosed asthma, were enrolled. In addition, 270 reference families were randomly selected from the same area. The following data were collected from each participant: 1) a questionnaire to assess respiratory symptoms, history of disease, smoking and alcohol consumption, and occupational and environmental exposure; 2) a standardized spirometry test to measure forced expiratory volume in 1s (FEV1) and forced vital capacity (FVC); 3) airway methacholine (MTCH) challenge test with the following 5 combinations of number of breaths and methacholine concentration in sequential order: 1 breath of 1 mg/mL, 1 breath of 5 mg/mL, 4 breaths of 5 mg/mL, 1 breath of 25 mg/mL, and 4 breaths of 25 mg/mL; the test was terminated at the dose that produced a ≥20% drop in FEV1 from the baseline (PD20), or at the final dose if PD20 was not observed; BHR was defined as a PD20 was observed during the test; and BHR asthma was defined as physician-diagnosed asthma combined with BHR; 4) skin prick test with following antigens applied: cockroach, house dust, mixed trees, mixed grasses, tobacco leaf, polyvalent molds, mite (*Dermatophagoides farinae*), artemisia, silk, and mite (*Dermatophagoides pteronyssinus*); an histamine (0.5%) and a saline control were also applied; an antigen-induced wheal size >2 mm of the saline control value was considered positive; a positive skin test was defined as at least one positive reaction to the applied antigens was observed; and 5) measurement of serum IgE levels.

The 1183 samples in this study were in two groups based on their phenotypes. The subjects in the first group (n = 543) had physician-diagnosed asthma and one or more of the following conditions: 1) an observed PD20 at or before the third dose in the MTCH challenge test; 2) total serum IgE in the top quartile of the population IgE distribution; and 3) total number of positive reactions to the 10 allergens in the skin prick test in the top quartile of the population distribution. The subjects in the second group (n = 640) did not have physician diagnosed asthma and met one or more of the following conditions: 1) PD20 not observed during the MTCH challenge test; 2) total serum IgE in the bottom quartile of the population IgE distribution; and 3) total number of positive reactions to the 10 allergens in the bottom quartile of the population distribution. The 1183 samples were unrelated with each other. The Human Subjects Committees at both the Harvard School of Public Health and the Anhui Medical University approved the study. Written informed consent was explained to, read and signed by each participant.

### SNP Selection and Genotyping

DNA was extracted from leukocytes in peripheral blood using standard techniques. The aim of the current study was to replicate the previously identified associations. Therefore, for PHF11 we selected two SNPs (rs2247119 and rs1046295) that produced the strongest association in the previous report [7]. The previous association in DPP10 peaked at a microsatellite marker D2S308 in the initial exons [2]. Instead of relying on the microsatellite markers, we selected five tag SNPs (rs10192393, rs1430092, rs1430090, rs6737251, and rs7580359) that tagged a 50-kb region flanking the initial exons of DPP10. SNP rs1632947 in the promoter region of HLA-G produced the strongest association in the previous report [3]. However this SNP was not genotyped in the HapMap project. Instead, we selected two tag SNPs (rs2247119 and rs1046295) that tagged the whole 3-kb promoter region of HLA-G. All the tagging selections were done using the HapMap Chinese (CHB) genotype data [21] and the SNPbrowser (Applied Biosystems, Foster City, CA), and tagged all the SNPs with ≥5% minor allele frequency (MAF) in the region of interest with a minimal pairwise r^2 ^of 0.8. Genotyping was done using Taqman genotyping assays designed and manufactured by Applied Biosystems. The genotype call rate of the 9 selected SNPs ranged from 95.0% to 98.8%. A random 5% of the samples were independently repeated to confirm the genotyping results. The concordance of these duplicated samples was 100%.

### Statistical Analyses

In our association analyses, we examined the associations of polymorphisms in the three genes to asthma and asthma-related phenotypes according to previous study results [1-3,7,9-11], i.e. total IgE levels and skin prick test for PHF11, and MTCH challenge test for DPP10 and HLA-G. Among these phenotypes, the log10 transformed IgE was quantitative; asthma status, MTCH challenge test results (BHR), and skin prick test results were treated as dichotomous. Since it has been shown that combining asthma-related phenotypes with physician's diagnosis of asthma led to a stronger association to the asthma candidate genes [4,22], in the current study, dichotomous asthma-related phenotypes with a single trait p value less than 0.1 were further combined with asthma status to create more extreme and genetically homogeneous cases and controls, so that all cases were asthmatic with the positive asthma-related phenotype, while all controls were non-asthmatic with the negative asthma-related phenotype.

We used a *t *test and a χ^2 ^test to test for differences in distributions of quantitative traits and categorical variables between asthmatic and non-asthmatic samples, respectively. The same methods were used to test for differences in distributions of those variables among different genotypes as well. We examined each SNP for Hardy-Weinberg equilibrium (HWE) in non-asthmatic samples using a χ^2 ^test. We used Haploview [23] to calculate LD between the SNPs among the same gene and to infer haplotype blocks. We performed single-SNP association tests on asthma and asthma-related phenotypes using logistic regression or linear regression analyses under different genetic models (additive or dominant) with the adjustment for age, gender, height, weight, BMI and smoking status. We also performed haplotype association tests using Haplo.Stats [24]. All the analyses were performed by using the statistical software program R http://www.r-project.org. To account for the multiple tests we made with various SNPs, haplotypes and phenotypes in the study, a permutation-based procedure was used to estimate empirical study-wide type I error rate of 0.05 [25]. In brief, genotypes of all SNPs were randomly permuted 10000 times. After each permutation, same single-SNP and haplotype analyses with multiple phenotypes were performed and the minimum p value among these tests was recorded. As a result, 10000 minimum p values were created to represent their null distribution. The study-wide type I error rate of 0.05 was then estimated as the 500th (10000 × 0.05) smallest minimum p value.

## Results

9 SNPs across the top hit regions in PHF11, DPP10, and HLA-G were selected to replicate the previously identified associations. We tested these SNPs for association in 1183 unrelated subjects selected from our study population. The phenotypic characteristics of the study samples are summarized in Table 1. There were only slight differences between asthmatic and non-asthmatic samples in terms of age, height, weight and BMI. All SNPs were in HWE, and their MAFs are listed in Table 2.

**Table 1:** Basic phenotypic information of 1183 population-based samples.

	Asthmatic Samples			Non-asthmatic Samples		
	
Variables	N	Mean	SD	N	Mean	SD
Age (yr)*	543	28.5	16.1	640	31.0	16.7
Gender (male%)	543	53.2	-	640	49.2	-
Height (m)*	543	1.50	0.16	639	1.54	0.14
Weight (kg)*	543	44.4	13.6	639	48.6	12.8
BMI (kg/m^2^) ^a^*	543	19.0	3.19	639	20.1	3.15
Smoke (yes%)	543	25.8	-	640	25.8	-
Education (elementary school or lower%)	367	42.8	-	492	43.7	-
Occupation (farmer%)	365	69.0	-	493	65.7	-
Dust/gas/fume Exposure (yes%)	366	11.7	-	492	10.8	-
Asthma (yes%)*	543	100	-	640	0	-
FEV1 (L)^b^*	526	2.20	0.84	623	2.81	0.89
FVC (L)^c^*	526	3.22	1.14	623	3.51	1.09
% Predicted FEV1*	526	83.2	18.9	623	94.0	10.0
% Predicted FVC	526	92.3	11.9	623	93.5	9.1
BHR (yes%) ^d^*	432	87.3	-	546	24.0	-
Total Log IgE (IU)*	320	6.11	1.40	296	2.89	1.77
Positive Skin Reactions (yes%)^e^*	427	81.0	-	590	32.2	-

^a^: Body mass index.

^b^: Forced expiratory volume in 1s.

^c^: Forced vital capacity.

^d^: Bronchial hyperresponsiveness.

^e^: Mean wheel size for the total 10 applied antigens.

* P < 0.05 between two groups.

**Table 2:** Minor allele frequencies of the 9 studied SNPs.

Gene	SNP	Allele	**MAF**^**a**^
PHF11	rs2247119	T->C	0.422
	rs1046295	G->A	0.496
DPP10	rs10192393	T->C	0.054
	rs1430092	C->T	0.089
	rs1430090	T->G	0.395
	rs6737251	C->T	0.255
	rs7580359	C->T	0.268
HLA-G	rs1632949	A->G	0.308
	rs1736935	C->T	0.415

^a^: Minor allele frequency (MAF) was calculated from 640 non-asthmatic samples.

*PHF11 *In our analyses, the rs2247119 C allele tended to increase the risk of positive skin reactions to antigens, although it was not statistically significant (Table 3). However, compared to the **TG **haplotype constructed by rs2247119 and rs1046295, the **CG **haplotype was nominally associated with a 2.05 times higher risk of skin reactions to antigens (nominal P = 0.023) (Table 4). Further combining skin reactions and asthma status did not yield more significant association results (Table 3 and 4). After the adjustment for multiple testing, the study-wide significance level was estimated as 0.0041 by the permutation procedure. Compared to this level, the haplotype association was not significant.

**Table 3:** Associations between SNPs in PHF11, DPP10, HLA-G and asthma or asthma-related phenotypes, adjusted for age, gender, height, weight, BMI and smoking status.

Gene	Phenotype	SNP	Genotype	Negative N (%)	Positive N (%)	OR (95% CI)	Nominal P Value
PHF11	SPT^a^	rs2247119	TT	167 (35.9)	161 (30.8)	1	
			CT	213 (45.8)	263 (50.3)	1.27 (0.95-1.67)	0.10
			CC	85 (18.3)	99 (18.9)	1.14 (0.79-1.65)	0.48
				Dominant Model		1.23 (0.94-1.61)	0.13
							
	SPT + Asthma ^b^	rs2247119	TT	138 (35.8)	109 (32.2)	1	
			CT	179 (46.5)	169 (49.9)	1.18 (0.84-1.65)	0.35
			CC	68 (17.7)	61 (18.0)	1.08 (0.69-1.67)	0.74
				Dominant Model		1.15 (0.84-1.58)	0.39
							
DPP10	MTCH Challenge^c^	rs10192393	TT	404 (92.0)	393 (86.2)	1	
			CT	34 (7.7)	62 (13.6)	1.91 (1.21-3.03)	**0.0059**
			CC	1 (0.2)	1 (0.2)	1.46 (0.09-23.56)	0.79
				Dominant Model		1.90 (1.20-3.00)	**0.0059**
							
	MTCH Challenge + Asthma ^d^	rs10192393	TT	360 (92.3)	284 (85.8)	1	
			CT	29 (7.4)	46 (13.9)	2.04 (1.22-3.42)	**0.0070**
			CC	1 (0.3)	1 (0.3)	1.66 (0.10-27.13)	0.72
				Dominant Model		2.03 (1.22-3.37)	**0.0067**
							
HLA-G	MTCH Challenge	rs1736935	CC	150 (32.6)	191 (37.5)	1	
			CT	227 (49.3)	241 (47.3)	0.82 (0.61-1.09)	0.17
			TT	83 (18.0)	77 (15.1)	0.73 (0.49-1.07)	0.10
				Additive Model		0.85 (0.70-1.02)	0.079
							
	MTCH Challenge + Asthma	rs1736935	CC	136 (33.9)	152 (39.3)	1	
			CT	192 (47.9)	181 (46.8)	0.84 (0.61-1.15)	0.27
			TT	73 (18.2)	54 (14.0)	0.64 (0.41-0.99)	**0.044**
				Additive Model		0.81 (0.65-0.99)	**0.043**

^a^: Skin prick test. A positive skin prick test was defined as at least one antigen-induced wheal size >2 mm of the saline control value.

^b^: Combined asthma status and skin prick test. All cases were asthmatic with a positive skin test result, while all controls were non-asthmatic with a negative skin test result.

^c^: Airway methacholine challenge test. Negative and positive responses were defined as no PD_20 _observed and a PD_20 _observed in the test, respectively.

^d^: Combined asthma status and methacholine challenge test. All cases were asthmatic with a positive challenge test result, while all controls were non-asthmatic with a negative challenge test result.

**Table 4:** Associations between haplotypes in PFH11, DPP10, HLA-G and asthma-related phenotypes, adjusted for age, gender, height, weight, BMI and smoking status.

Gene	Phenotype	**Haplotype**^**a**^			Negative	Positive	**OR (95% CI)**^**b**^	Nominal P value
							
					Frequency (%)	Frequency (%)		
		rs2247119		rs1046295				
						
PHF11	SPT^c^	T		G	47.9	46.5	1	
		C		A	38.9	39.7	1.06 (0.81-1.40)	0.67
		T		A	11.3	9.6	0.85 (0.60-1.19)	0.34
		C		G	2.0	4.1	2.05 (1.11-3.81)	**0.023**
								
	SPT + Asthma ^d^	T		G	48.4	47.7	1	
		C		A	38.5	39.0	1.04 (0.75-1.44)	0.80
		T		A	10.9	9.6	0.88 (0.59-1.32)	0.55
		C		G	2.1	3.7	1.59 (0.78-3.25)	0.20
								
		rs10192393		rs1430092				
						
DPP10	MTCH Challenge^e^	T		C	88.2	84.4	1	
		T		T	7.9	8.7	1.25 (0.86-1.82)	0.24
		C		C	3.9	6.8	1.99 (1.25-3.17)	0.0040*
								
	MTCH Challenge + Asthma ^r^	T		C	88.1	83.2	1	
		T		T	8.1	9.8	1.33 (0.88-2.01)	0.17
		C		C	3.8	7.0	2.15 (1.28-3.62)	0.0041*
								
		rs1430090	rs6737251	rs7580359				
						
	MTCH Challenge	G	C	C	38.7	39.2	1	
		T	C	C	34.6	35.5	0.98 (0.74-1.31)	0.90
		T	T	T	25.3	23.6	0.88 (0.66-1.16)	0.36
		G	C	T	1.4	1.7	1.23 (0.54-2.78)	0.62
								
		rs1632949		rs1736935				
						
HLA-G	MTCH Challenge	A		T	43.2	38.8	1	
		G		C	31.5	32.1	1.15 (0.93-1.44)	0.20
		A		C	25.4	29.0	1.24 (0.99-1.56)	0.060
								
	MTCH Challenge + Asthma	A		T	42.6	37.5	1	
		G		C	32.2	30.8	1.11 (0.87-1.43)	0.40
		A		C	25.2	31.7	1.40 (1.09-1.80)	**0.0086**

^a^: Haplotypes with frequency ≥0.01 were considered.

^b^: The main effects of haplotypes were in dominant, dominant and additive models for PHF11, DPP10 and HLA-G, respectively.

^c^: Skin prick test. A positive skin prick test was defined as at least one antigen-induced wheal size >2 mm of the saline control value.

^d^: Combined asthma status and skin prick test. All cases were asthmatic with a positive skin test result, while all controls were non-asthmatic with a negative skin test result.

^e^: Airway methacholine challenge test. Negative and positive responses were defined as no PD_20 _observed and a PD_20 _observed in the test, respectively.

^f^: Combined asthma status and methacholine challenge test. All cases were asthmatic with a positive challenge test result, while all controls were non-asthmatic with a negative challenge test result.

*: The associations remained significant (adjusted P ≤ 0.05) after the adjustment for multiple testing.

**Figure 1:**
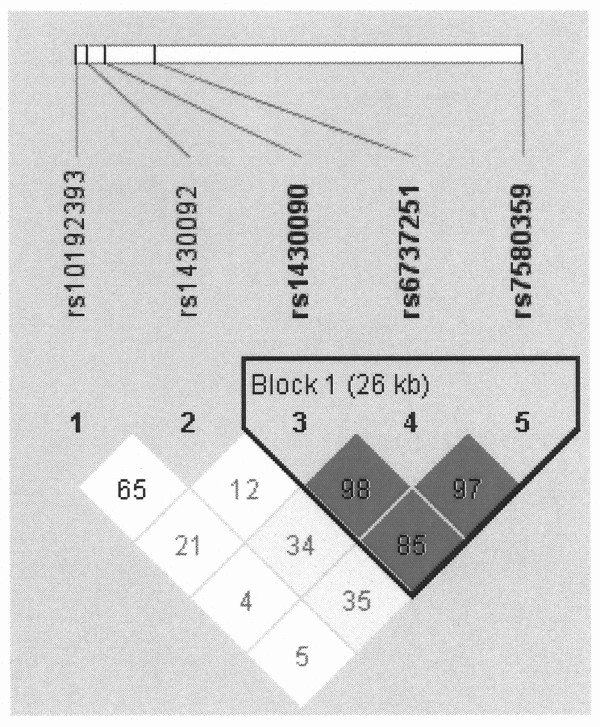
**LD structures of 5 SNPs on DPP10**. D' values are listed in each cell and the haplotype block is defined by the solid spine of LD.

*DPP10 *Among the five studied SNPs, rs10192393 produced the strongest association signal (Table 3 and additional file 1 - Supplemental Table E2). Compared with the rs10192393 T/T homozygotes, individuals with C/C or C/T genotype had a 1.90 times higher risk of BHR (nominal P = 0.0059) and a 2.03 times higher risk of BHR asthma (nominal P = 0.0067) (Table 3). Among the five SNPs, rs1430090, rs6737251 and rs7580359 were in a haplotype block defined by the solid LD spine (Figure 1) [23]. We further explored the associations with haplotypes constructed by the two SNPs outside the block and by the three SNPs inside the block, respectively. As shown in Table 4, compared to subjects with the **TC **haplotype constructed by rs10192393 and rs1430092, ones with the **CC **haplotype had a 1.99 times higher risk of BHR (nominal P = 0.0040) and a 2.15 times higher risk of BHR asthma (nominal P = 0.0041). No significant association was found for haplotypes inside the block. Compared to the study-wide significance level of 0.0041, only the haplotype associations remained significant.

*HLA-G *In single-SNP analyses, compared with the rs1736935 C/C homozygotes, individuals with T/T genotype had a lower risk of BHR asthma (OR = 0.64, nominal P = 0.044) (Table 3). Under the additive genetic model, we estimated that each additional copy of the T allele reduced the risk of BHR asthma by 19% (OR = 0.81; nominal P = 0.043). In haplotype analyses, we found that compared to the **AT **haplotype constructed by rs1632949 and rs1736935, each additional copy of the **AC **haplotype had a 1.40 times higher risk of BHR asthma (nominal P = 0.0086) (Table 4). However, compared to the study-wide significance level of 0.0041, all the associations became not significant.

## Discussion

Because asthma is a clinical syndrome and there are no clinical benchmarks, several asthma-related phenotypes are used to help the diagnosis. Since these phenotypes are objective, quantitative, and of less genetic heterogeneity, they are often used in genetic studies to search for the causal genetic variants for asthma [26]. Evidence has shown that combining asthma-related phenotypes with physician's diagnosis of asthma led to a stronger association to the asthma candidate genes, which may shed light on the different molecular pathways underlying asthma [4,22]. In our current study, we selected study samples by combining asthma affectation status and three asthma-related phenotypes, including total serum IgE levels, bronchial responsiveness test, and skin prick test. This study design provided more power to examine various mechanisms of asthma. We observed that combining BHR with asthma produced stronger genetic effects (odds ratios) in both DPP10 single SNP and haplotype analyses as compared to BHR alone. This indicates that the gene may participate in asthma susceptibility through the mechanisms of BHR.

Our results confirm previous associations of DPP10 and asthma or asthma-related phenotype, but differ in the details. In the positional cloning study of DPP10, Allen *et al*. demonstrated that the strongest association signal to asthma came from the allele 3 of a microsatellite marker D2S308 in a population containing three Caucasian cohorts [2]. Studies have shown that DPP10 is strongly expressed in central neural system and is a modulator of voltage-gated K+ channel inactivation [2,11]. This indicates that DPP10 may be important in neural regulation of airway smooth muscle and control of airway reactivity, which may explain our finding that the haplotype containing the SNP rs10192393 in DPP10, 4 kb upstream of D2S308, was associated with BHR and BHR asthma. For PHF11 and HLA-G, the associations with asthma or asthma-related phenotypes were only significant at the nominal level, and became not significant after the adjustment for multiple testing. However, these results might still be of interest. Zhang *et al*. identified the locus at the PHF11 gene as a determinant of serum IgE levels [1]. However, we did not find any association between PHF11 and IgE in our Chinese cohort even at the nominal significance level. Recently, Jang *et al*. provided evidence for an association between rs2247119 and rs1046295 in PHF11 and atopic dermatitis in a young Australian cohort [7]. In their study, the T allele from rs2247119 and G allele from rs1046295 were over-transmitted to children with atopic dermatitis. The correlation between these two SNPs in Chinese population (r^2 ^= 0.53) is moderate compared with that in Australian population (r^2 ^= 0.92) [7]. In our current study, however, we found that the haplotype containing the C allele of rs2247119 (rather than the T allele) and the G allele of rs1046295 was nominally associated with an increased risk of positive skin reactions to antigens. Nicolae *et al*. identified HLA-G as asthma and BHR susceptibility gene in two Chicago cohorts by examining pairwise combination of SNPs [3]. The association of SNP rs1632947 in the promoter region of HLA-G with BHR was confirmed in the Hutterite families, where the C allele was over-transmitted to children with BHR. In our current study, we found that the genotype containing the C allele at rs1736935, one of the two selected tag SNPs 215 bp upstream of rs1632947, and the haplotype containing the rs1736935 C allele were nominally associated with an increased risk of BHR asthma. It is probably due to the small effect sizes of the genetic variants and lack of power in the current study that the associations of PHF11 and HLA-G with asthma or asthma-related phenotypes failed to reach the study-wide significance level. Therefore, studies with larger sample sizes can be conducted to further evaluate the nominal associations of PHF11 and HLA-G identified in the current study.

The population-based study design is a common strategy used in genetic studies of complex diseases. Population homogeneity is desirable to avoid population stratification. Our study samples are relatively homogenous. All of them are from a rural area of Anqing, China, a region known for a historically stable population. Not only is there a predominant singular ethnic group, but there exists a uniformity of occupation (farming) and lifestyle throughout Anqing. This population homogeneity minimizes the potential of population stratification that may lead to false-positive associations. Also, in a previous asthma candidate gene study, we tested 119 SNPs from 105 genes for associations with asthma using 170 asthmatic cases and 347 controls selected from the same 3022 families where the current study subjects were sampled [27]. We applied STRUCTURE2.1 to assess the presence of population substructures within our previous study samples. A set of 111 SNPs after excluding potentially associated ones (p < 0.05 in association tests) was used in the analyses. STRUCURE2.1 showed that a single substructure had the highest likelihoods among all possible number of substructures in the samples, which indicated that the samples in our previous study were homogeneous. Even though the study samples were different between the current and previous studies, they were actually selected from the same population pool in the same geographic region, so the conclusion of no population stratification based on one study can be generalized to the other study.

In the current study, several demographic factors, including age, height, weight, and BMI, were slightly imbalanced between asthmatic and non-asthmatic samples. To minimize their potential confounding effects, we included these factors as covariates in all the analyses. Furthermore, we examined the distributions of these factors among samples with different genotypes of studied SNPs and found no significant differences. This indicated that there were no correlations between studied SNPs and four above-mentioned factors. Therefore, the results were not confounded by age, height, weight, and BMI. Also, gender did not bias the results since it was balanced between asthmatic and non-asthmatic samples. Moreover, the genetic effects were similar in gender-specific (males and females) analyses and there was no significant interaction between gender and the DPP10 haplotype in the log-likelihood-ratio test (data not shown). Note that our study subjects include both children and adults. Although evidence showed that childhood and adult asthma differed in several parameters [28,29], the associations between PHF11, DPP10, HLA-G and asthma or asthma-related phenotypes were found in both groups [1-3,7]. Therefore, these genes may play roles in the common pathway of childhood and adult asthma. Further subgroup (subjects aged <21 yrs and ≥21 yrs) analyses were performed and similar associations were detected in both younger and older groups; also, there was no significant interaction between age and the DPP10 haplotype by the log-likelihood-ratio test (data not shown). This suggests that age may have a minimal effect on the associations.

## Conclusion

In conclusion, our study was the first one to demonstrate that DPP10 is associated with BHR and BHR asthma in a Chinese population. Our study provides supportive evidence to the previously reported associations between the DPP10 gene and asthma or asthma-related phenotypes in Caucasian populations.

## Competing interests

The authors declare that they have no competing interests.

## Authors' contributions

HZ conceived of the study, participated in its design, performed statistical analysis and drafted the manuscript. XH participated in the design of the study. SJ and HD performed the genotyping. XX and XX conceived of the study, participated in its design and coordination and helped to draft the manuscript. All authors read and approved the final manuscript.

## Pre-publication history

The pre-publication history for this paper can be accessed here:

http://www.biomedcentral.com/1471-2350/10/123/prepub

## Supplementary Material

Additional file 1 **Supplemental Tables**. Supplemental Table E1 and E2 showing the non-significant association results. Click here for file
