# Supplementary material for: Analyses of associations between three positionally cloned asthma candidate genes and asthma or asthma-related phenotypes in a Chinese population
Source: BMC Med Genet. 2009 Dec 1;10:123. doi: 10.1186/1471-2350-10-123 (PMC2799396; doi:10.1186/1471-2350-10-123)
Supplement: Additional file 1 — Supplemental Tables. Supplemental Table E1 and E2 showing the non-significant association results. [file 1471-2350-10-123-S1.DOC]

**Additional file 1**

**Analyses of associations between three positionally cloned asthma candidate genes and asthma or asthma-related phenotypes in a Chinese population**

Huanyu Zhou^1^, Xiumei Hong^2^, Shanqun Jiang^3^, Hongxing Dong^3^, Xiping Xu^2^, Xin Xu^1^*

^1^Program for Population Genetics, Harvard School of Public Health, Boston, Massachusetts, USA

^2^Division of Epidemiology and Biostatistics, School of Public Health, University of Illinois at Chicago, Chicago, Illinois, USA

^3^Institute of Biomedicine, Anhui Medical University, Hefei, Anhui, PR China

* Corresponding author

E-mail addresses:

Huanyu Zhou: [huanyuzhou@hrca.harvard.edu](mailto:huanyuzhou@hrca.harvard.edu)

Xiumei Hong: [xiumei.hong@gmail.com](mailto:xiumei.hong@gmail.com)

Shanqun Jiang: [shanqun@gmail.com](mailto:shanqun@gmail.com)

Hongxing Dong: [rstar126900@gmail.com](mailto:rstar126900@gmail.com)

Xiping Xu: [xipingxu18@gmail.com](mailto:xipingxu18@gmail.com)

Xin Xu: [xin_xu@harvard.edu](mailto:xin_xu@harvard.edu)

Supplemental Table E1. Non-significant associations between SNPs in PHF11 and quantitative IgE, adjusted for age, gender, height, weight, BMI and smoking status.

| **Gene** | **Phenotype** | **SNP** | **Genotype** | **N** | **Beta (95% CI)** |
| --- | --- | --- | --- | --- | --- |
| PHF11 | Log IgE | rs2247119 | TT | 188 | 0 |
|  |  |  | CT | 298 | 0.06 (-0.12-0.24) |
|  |  |  | CC | 116 | 0.05 (-0.17-0.28) |
|  |  |  |  |  |  |
|  |  | rs1046295 | GG | 151 | 0 |
|  |  |  | AG | 296 | 0 (-0.19-0.19) |
|  |  |  | AA | 155 | 0.06 (-0.16-0.27) |

Supplemental Table E2. Non-significant associations between SNPs in PHF11, DPP10, HLA-G and dichotomous phenotypes, including asthma status, methacholine challenge test and skin prick test, adjusted for age, gender, height, weight, BMI and smoking status.

| **Gene** | **Phenotype** | **SNP** | **Genotype** | **Negative**  **N (%)** | **Positive**  **N (%)** | **OR (95% CI)** |
| --- | --- | --- | --- | --- | --- | --- |
| PHF11 | Asthma | rs2247119 | TT | 205 (33.1) | 187 (35.0) | 1 |
|  |  |  | CT | 299 (48.3) | 251 (46.9) | 0.91 (0.70-1.18) |
|  |  |  | CC | 115 (18.6) | 97 (18.1) | 0.91 (0.65-1.28) |
|  |  |  |  |  |  |  |
|  |  | rs1046295 | GG | 149 (24.0) | 143 (27.1) | 1 |
|  |  |  | AG | 321 (51.8) | 253 (47.9) | 0.82 (0.62-1.09) |
|  |  |  | AA | 150 (24.2) | 132 (25.0) | 0.93 (0.66-1.29) |
|  |  |  |  |  |  |  |
|  | SPT^a^ | rs1046295 | GG | 108 (23.2) | 137 (26.3) | 1 |
|  |  |  | AG | 246 (52.9) | 252 (48.5) | 0.82 (0.60-1.12) |
|  |  |  | AA | 111 (23.9) | 131 (25.2) | 0.91 (0.63-1.30) |
|  |  |  |  |  |  |  |
| DPP10 | Asthma | rs10192393 | TT | 549 (90.0) | 456 (88.7) | 1 |
|  |  |  | CT | 60 (9.8) | 57 (11.1) | 1.12 (0.75-1.65) |
|  |  |  | CC | 1 (0.2) | 1 (0.2) | 1.12 (0.08-18.74) |
|  |  |  | Dominant Model | | | 1.12 (0.76-1.64) |
|  |  |  |  |  |  |  |
|  |  | rs1430092 | CC | 522 (84.3) | 435 (81.2) | 1 |
|  |  |  | CT | 93 (15.0) | 97 (18.1) | 1.30 (0.95-1.79) |
|  |  |  | TT | 4 (0.6) | 4 (0.7) | 1.14 (0.28-4.65) |
|  |  |  | Dominant Model | | | 1.30 (0.95-1.77) |
|  |  |  |  |  |  |  |
|  |  | rs1430090 | TT | 231 (36.8) | 198 (36.9) | 1 |
|  |  |  | GT | 293 (46.7) | 258 (48.0) | 1.05 (0.81-1.37) |
|  |  |  | GG | 104 (16.6) | 81 (15.1) | 0.95 (0.66-1.35) |
|  |  |  |  |  |  |  |
|  |  | rs6737251 | CC | 353 (55.9) | 286 (53.7) | 1 |
|  |  |  | CT | 240 (38.0) | 216 (40.5) | 1.15 (0.90-1.47) |
|  |  |  | TT | 38 (6.0) | 31 (5.8) | 0.92 (0.55-1.53) |
|  |  |  |  |  |  |  |
|  |  | rs7580359 | CC | 343 (55.5) | 272 (50.8) | 1 |
|  |  |  | CT | 234 (37.9) | 223 (41.7) | 1.23 (0.96-1.58) |
|  |  |  | TT | 41 (6.6) | 40 (7.5) | 1.17 (0.73-1.87) |
|  |  |  |  |  |  |  |
|  | MTCH | rs1430092 | CC | 383 (84.7) | 388 (82.6) | 1 |
|  | Challenge ^b^ |  | CT | 65 (14.4) | 80 (17.0) | 1.25 (0.86-1.81) |
|  |  |  | TT | 4 (0.9) | 2 (0.4) | 0.35 (0.06-2.16) |
|  |  |  | Dominant Model | | | 1.19 (0.83-1.72) |
|  |  |  |  |  |  |  |
|  |  | rs1430090 | TT | 165 (36.2) | 173 (36.7) | 1 |
|  |  |  | GT | 221 (48.5) | 219 (46.5) | 0.92 (0.68-1.23) |
|  |  |  | GG | 70 (15.4) | 79 (16.8) | 1.10 (0.73-1.64) |
|  |  |  |  |  |  |  |
|  |  | rs6737251 | CC | 247 (53.7) | 270 (57.1) | 1 |
|  |  |  | CT | 188 (40.9) | 177 (37.4) | 0.91 (0.69-1.21) |
|  |  |  | TT | 25 (5.4) | 26 (5.5) | 0.79 (0.43-1.47) |
|  |  |  |  |  |  |  |
|  |  | rs7580359 | CC | 237 (52.8) | 258 (54.9) | 1 |
|  |  |  | CT | 181 (40.3) | 183 (38.9) | 0.98 (0.74-1.30) |
|  |  |  | TT | 31 (6.9) | 29 (6.2) | 0.76 (0.43-1.35) |
|  |  |  |  |  |  |  |
| HLA-G | Asthma | rs1632949 | AA | 286 (46.1) | 254 (47.9) | 1 |
|  |  |  | AG | 275 (44.4) | 236 (44.5) | 0.98 (0.77-1.26) |
|  |  |  | GG | 59 (9.5) | 40 (7.5) | 0.79 (0.50-1.22) |
|  |  |  |  |  |  |  |
|  |  | rs1736935 | CC | 202 (32.8) | 190 (36.3) | 1 |
|  |  |  | CT | 300 (48.7) | 250 (47.7) | 0.86 (0.66-1.12) |
|  |  |  | TT | 114 (18.5) | 84 (16.0) | 0.78 (0.55-1.11) |
|  |  |  |  |  |  |  |
|  | MTCH | rs1632949 | AA | 212 (45.7) | 237 (46.2) | 1 |
|  | Challenge |  | AG | 215 (46.3) | 224 (43.7) | 0.97 (0.74-1.27) |
|  |  |  | GG | 37 (8.0) | 52 (10.1) | 1.29 (0.80-2.08) |

^a^: Skin prick test. A positive skin prick test was defined as at least one antigen-induced wheal size >2 mm of the saline control value.

^b^: Airway methacholine challenge test. Negative and positive responses were defined as no PD_20_ observed and a PD_20_ observed in the test, respectively.
